# Supplementary material for: Exploring dynamic interactions of single nanoparticles at interfaces for surface-confined electrochemical behavior and size measurement
Source: Nat Commun. 2020 May 8;11:2307. doi: 10.1038/s41467-020-16149-0 (PMC7210955; doi:10.1038/s41467-020-16149-0)
Supplement: Supplementary file 1 — Supplementary Information [file 41467_2020_16149_MOESM1_ESM.pdf]

Supplementary Information

Exploring dynamic interactions of single nanoparticles at interfaces for surface-confined electrochemical behavior and size measurement

Ma *et al*.

Supplementary Figures

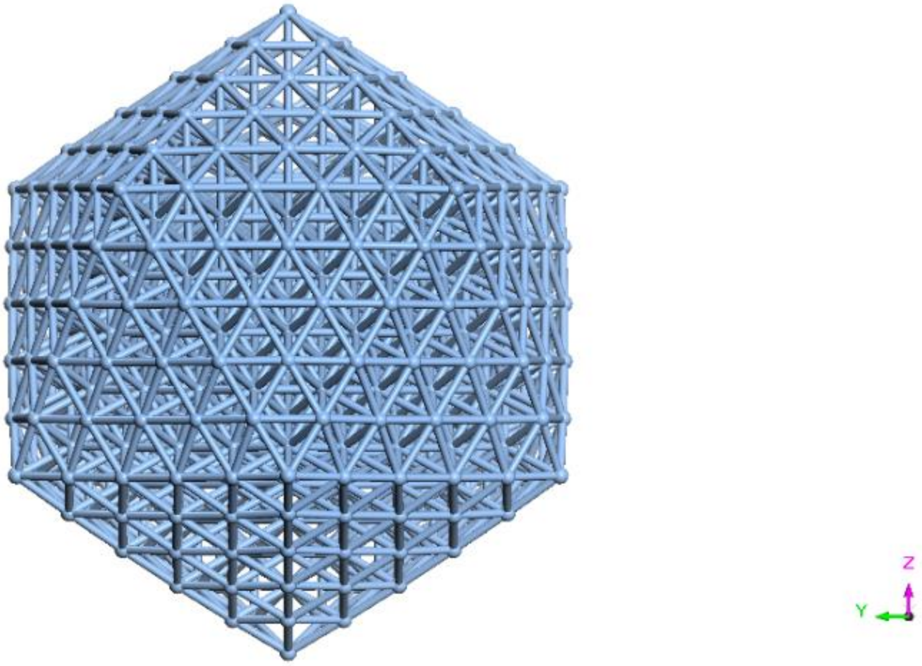

Supplementary Figure 1. Icosahedral structure of AgNPs. The structure is exhibiting 20 orderly arranged triangular-type (111) facets. Blue ball corresponds to Ag atoms.

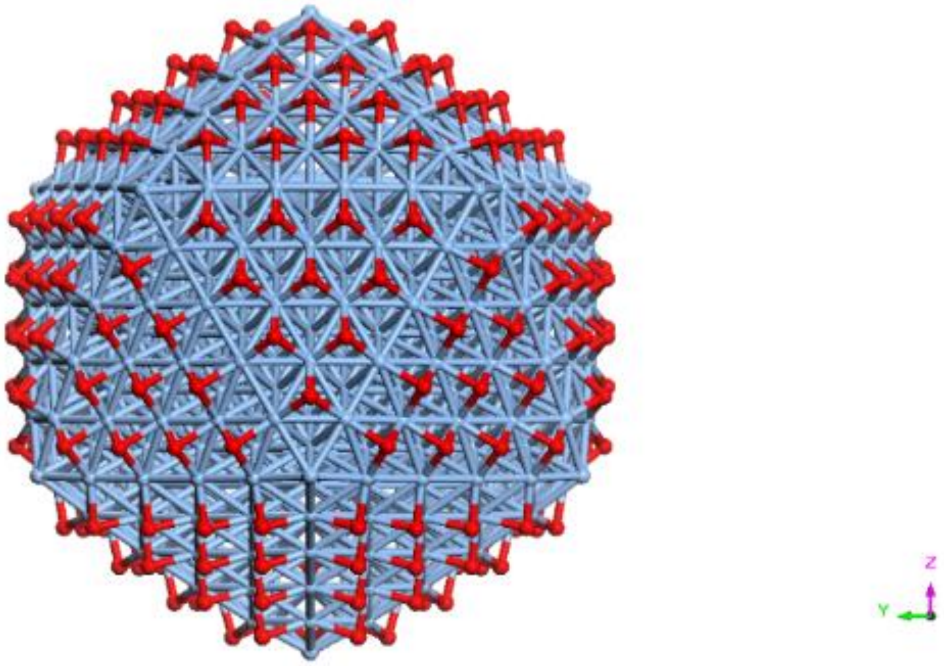

Supplementary Figure 2. Icosahedral structure of AgO_x NPs. Blue ball and red ball correspond to O and Ag atoms, respectively.

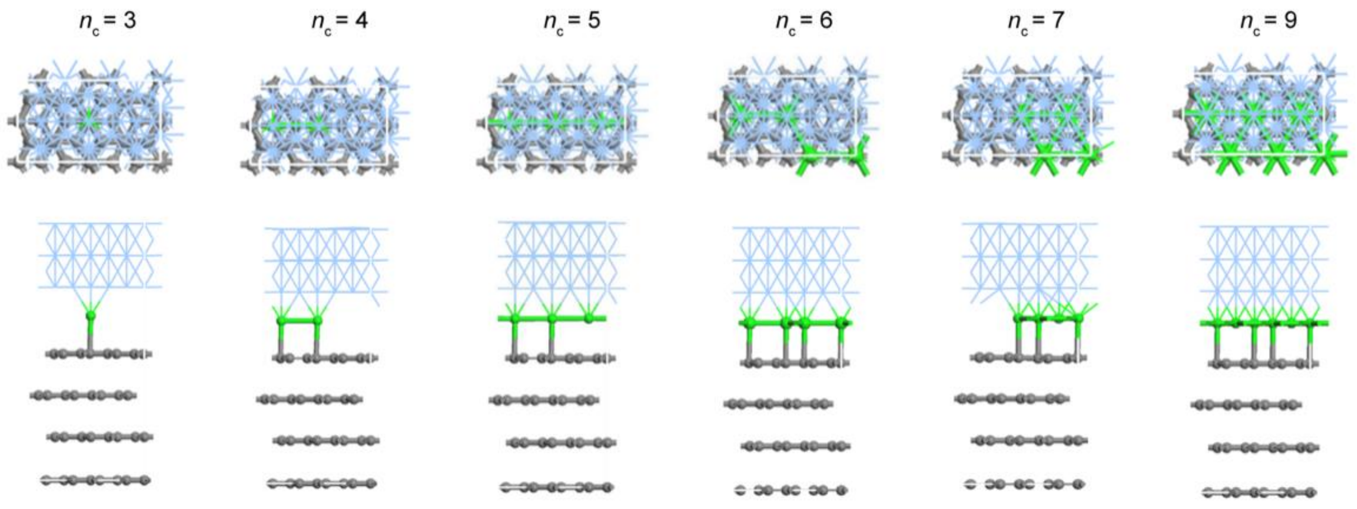

Supplementary Figure 3. Adsorption configurations of Ag atoms on C (002) basal plane. Top view (up) and side view (down) of the optimized adsorption configurations of Ag atoms with different coordination on C (002) basal plane for DFT calculation. Green ball/blue line and gray ball correspond to Ag and C atoms, respectively. Source data are provided as a Source Data file.

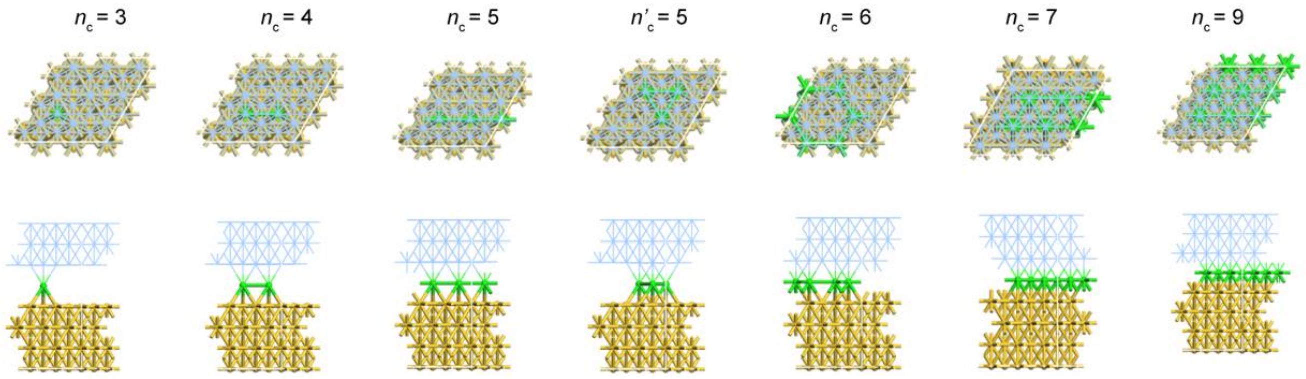

Supplementary Figure 4. Adsorption configurations of Ag atoms on Au (111) surface. Top view (up) and side view (down) of the optimized adsorption configurations of Ag atoms with different coordination on Au (111) surface for DFT calculation. Green ball/blue line and yellow ball correspond to Ag and Au atoms, respectively. Source data are provided as a Source Data file.

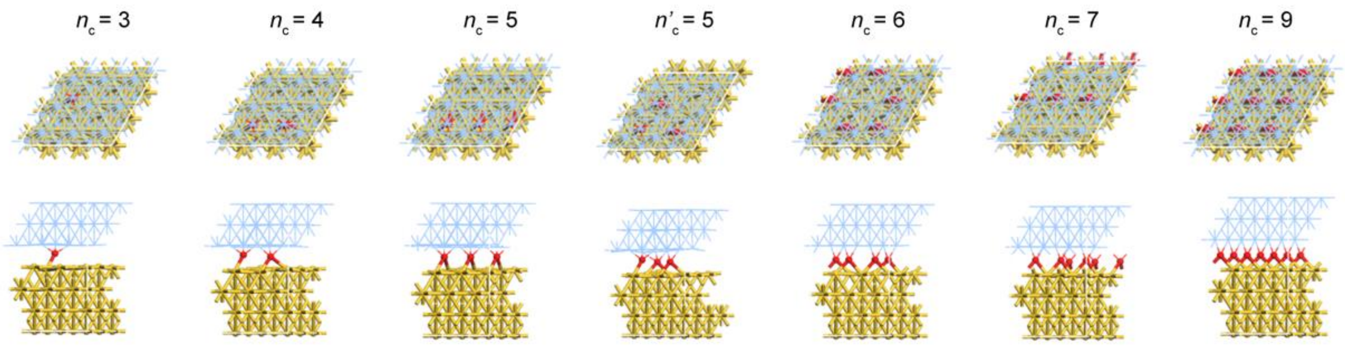

Supplementary Figure 5. Adsorption configurations of O atoms on Au (111) surface. Top view (up) and side view (down) of the optimized adsorption configurations of O atoms with different coordination on Au (111) surface for DFT calculation. Red ball, blue line and yellow ball correspond to O, Ag and Au atoms, respectively. Source data are provided as a Source Data file.

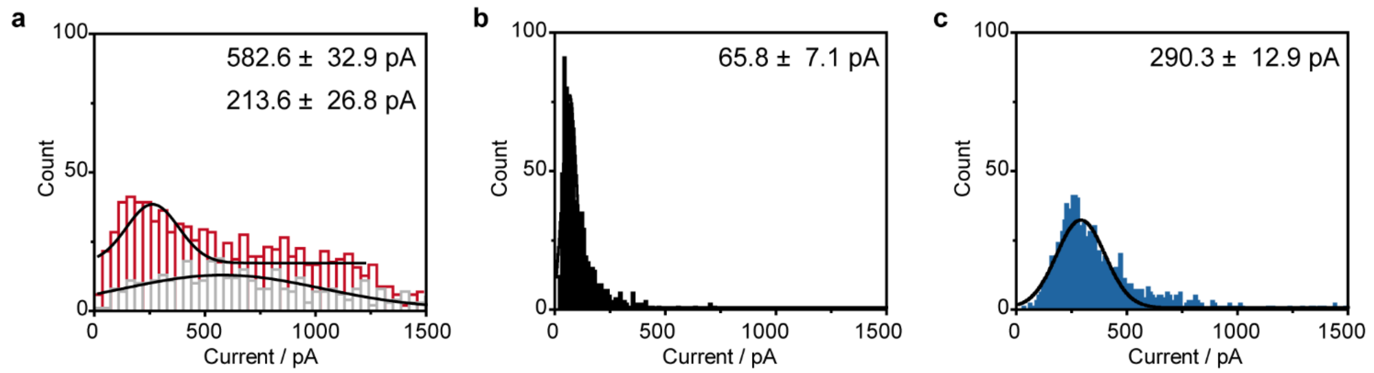

Supplementary Figure 6. Current distributions of individual AgNPs with the diameter of 34 nm. Current histograms of individual AgNPs at +600 mV vs Ag/AgCl (**a**) in neutral solution (25 mM PB, pH = 7.4) at the Au UME (12.5 μm in diameter; the gray bar and the red bar correspond to two current patterns arising from a single large peak and a spike with undulating terrain in Figure 2bii, respectively), (**b**) in neutral solution (25 mM PB, pH = 7.4, black bar) at the C UME (7 μm in diameter), and (**c**) in alkaline media (15 mM PB and 10 mM NaOH, pH = 11.4, blue bar) at the Au UME (12.5 μm in diameter). Black curves show Gaussian fits. The histograms were obtained from a large population of oxidation events of individual AgNPs (more than 1000 events). Source data are provided as a Source Data file.

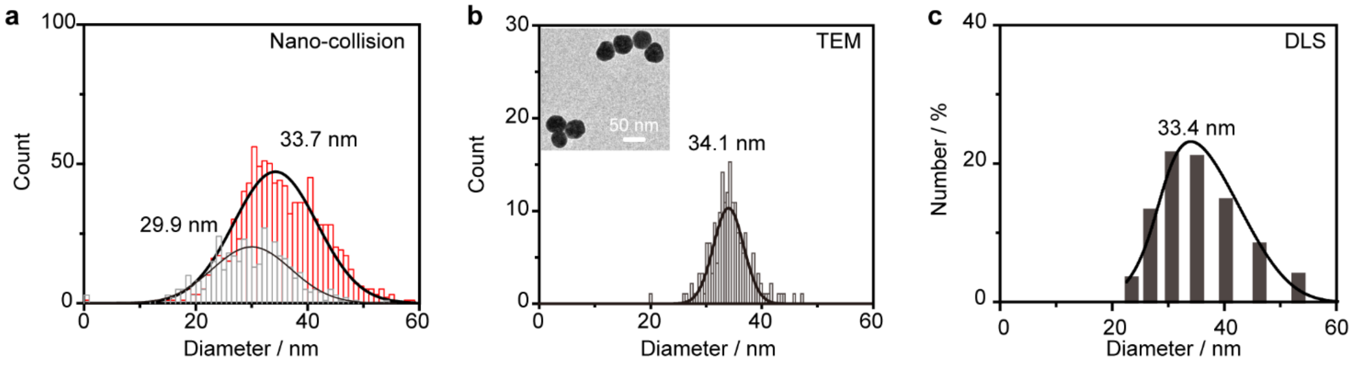

Supplementary Figure 7. Size distribution of 34 nm AgNPs. Histograms of AgNPs measured separately *via* nano-collision electrochemistry in neutral solution (25 mM PB, pH = 7.4) at the Au UME (12.5 μm in diameter) (**a**), TEM result (**b**) and DLS measurement (**c**). The gray bar and the red bar correspond to two current patterns arising from a single large peak and a spike with undulating terrain in Figure 2bii, respectively). Inset: TEM image of AgNPs. Source data are provided as a Source Data file.

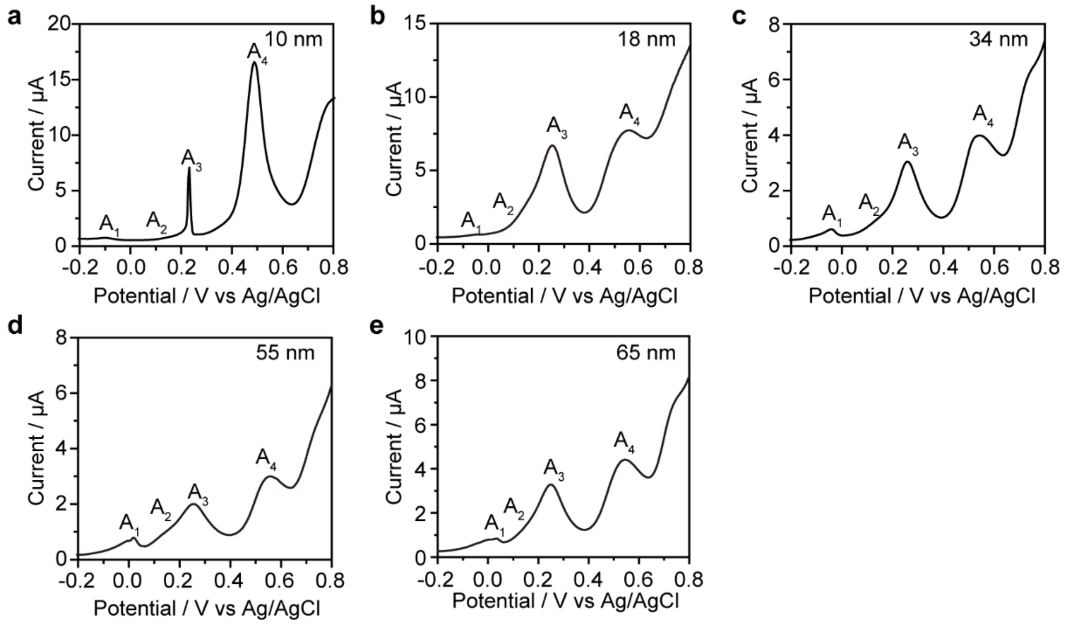

Supplementary Figure 8. Electrochemical oxidation of AgNPs in alkaline media. DPV for Au electrodes modified with (**a**) 10 nm, (**b**) 18 nm (**c**) 34 nm, (**d**) 55 nm, and (**e**) 65 nm AgNPs in alkaline media. The scan rate is 20 mV s^{-1}. Source data are provided as a Source Data file.

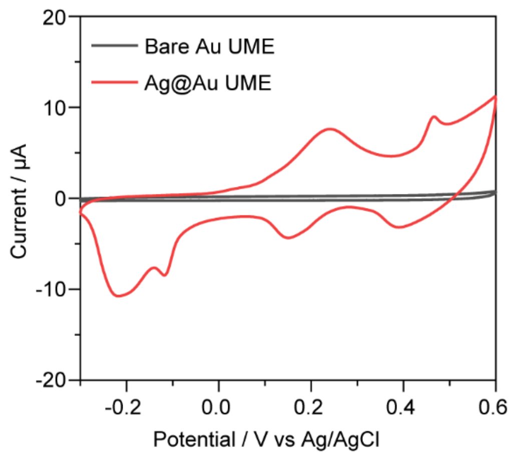

Supplementary Figure 9. Cyclic voltammograms for a bare Au electrode and a AgNPs modified Au electrode in alkaline media. The scan rate is 50 mV s^{-1}. Source data are provided as a Source Data file.

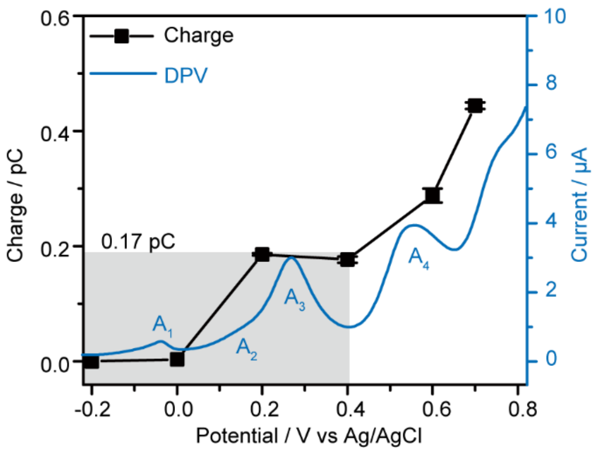

Supplementary Figure 10. Potential dependent integrated charge. Integrated charge of individual collision events as a function of different applied potentials for the electrochemical oxidation of 34 nm AgNPs in alkaline media. Corresponding DPV at a 34 nm AgNPs modified Au electrode in alkaline media. Source data are provided as a Source Data file.

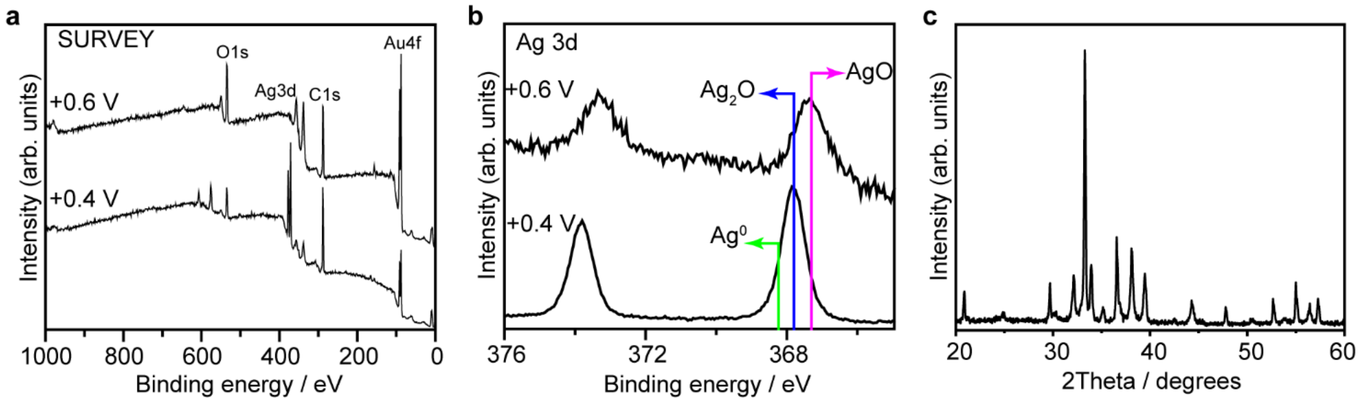

Supplementary Figure 11. Characterization of AgO formation. The XPS spectrum measured on the as-prepared silver sample. XPS survey spectrum (**a**) obtained from the oxide of AgNPs onto the gold substrate at the potential of +0.6 V and +0.4 V vs Ag/AgCl wire for 2 h in alkaline media (pH = 11.4), and the corresponding high-resolution XPS spectra of Ag3d (**b**). All XPS data were corrected for sample charging during X-ray irradiation using adventitious hydrocarbon referencing (C1s at 284.8 eV). (**c**) XRD pattern of 34 nm AgNPs on the surface of ITO electrode after anodic polarization for 1 h at +600 mV vs Ag/AgCl in alkaline media (15 mM PB and 10 mM NaOH). Source data are provided as a Source Data file.

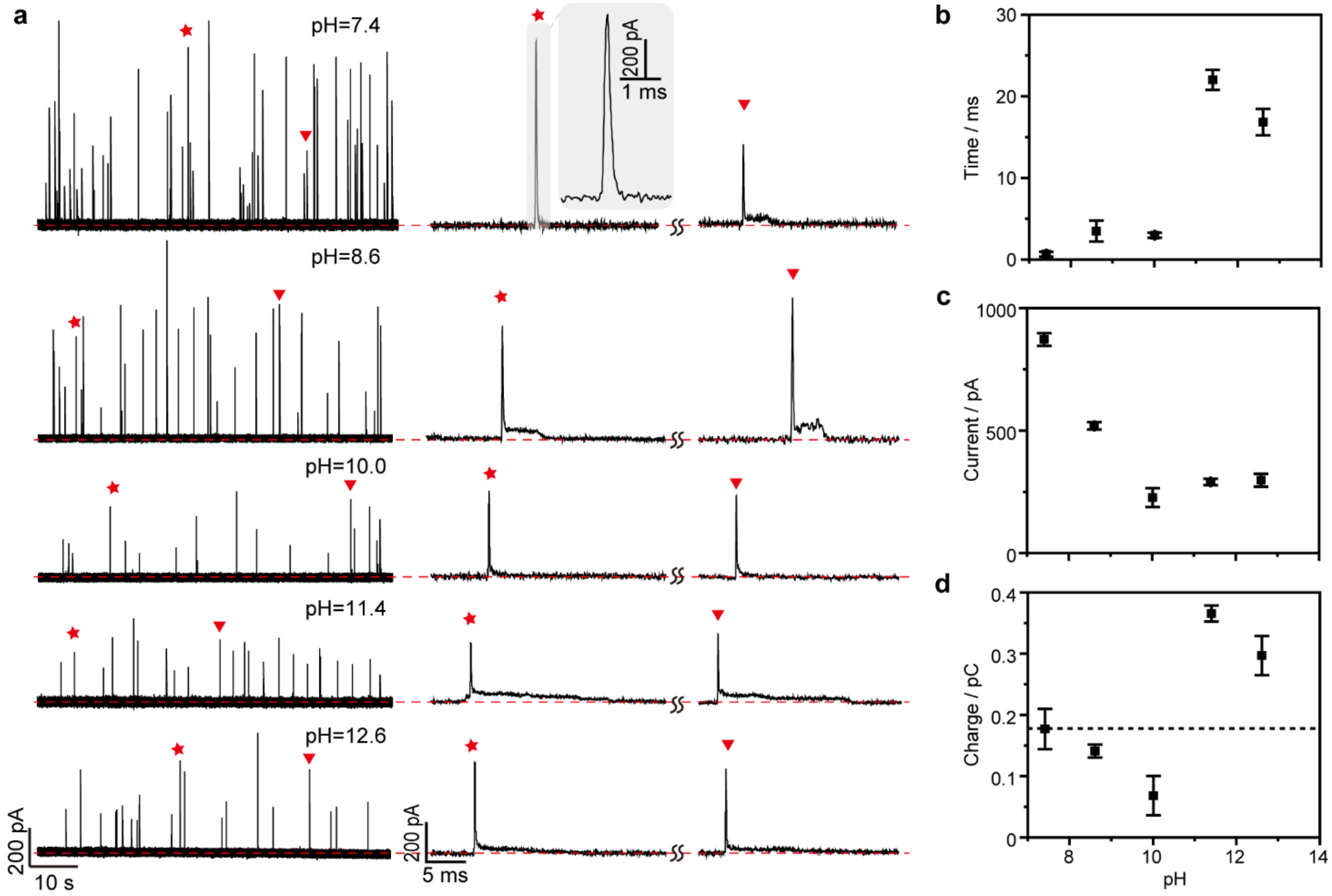

Supplementary Figure 12. pH effect on electrochemical oxidation of individual 34 nm AgNPs. (**a**) Chronoamperometric curves for the electrochemical oxidation of individual AgNPs with the same scale bar at +600 mV vs Ag/AgCl at a Au UME in PB solution at different pH and close-ups of the representative time-resolved current traces. Plots of the oxidation time (**b**), maximum current (**c**), and integrated charge (**d**) of 34 nm AgNPs in diameter at different pH. Error bars are the standard deviation from three independent experiments. Source data are provided as a Source Data file.

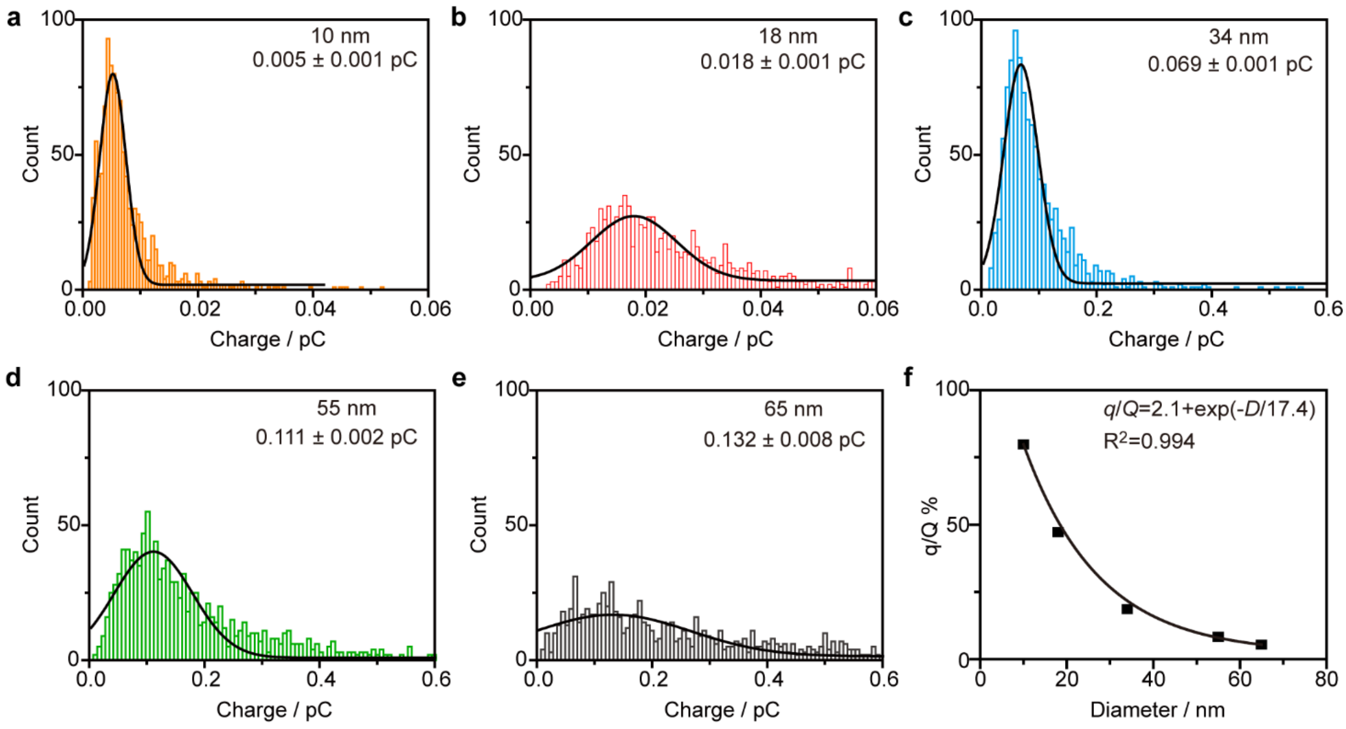

Supplementary Figure 13. Integrated charge of the initial spike. (**a-e**) Histograms of the integrated charge of the initial spike of AgNPs with different sizes (diameter: 10 nm, 18 nm, 34 nm, 55 nm and 65 nm). (**f**) Calibration curve of the ratio of the average faradaic charge from the initial spike to the average total charge (q/Q) vs the diameter of AgNPs. The black line was fitted by equation (10), and the correlation coefficient is 0.994. Source data are provided as a Source Data file.

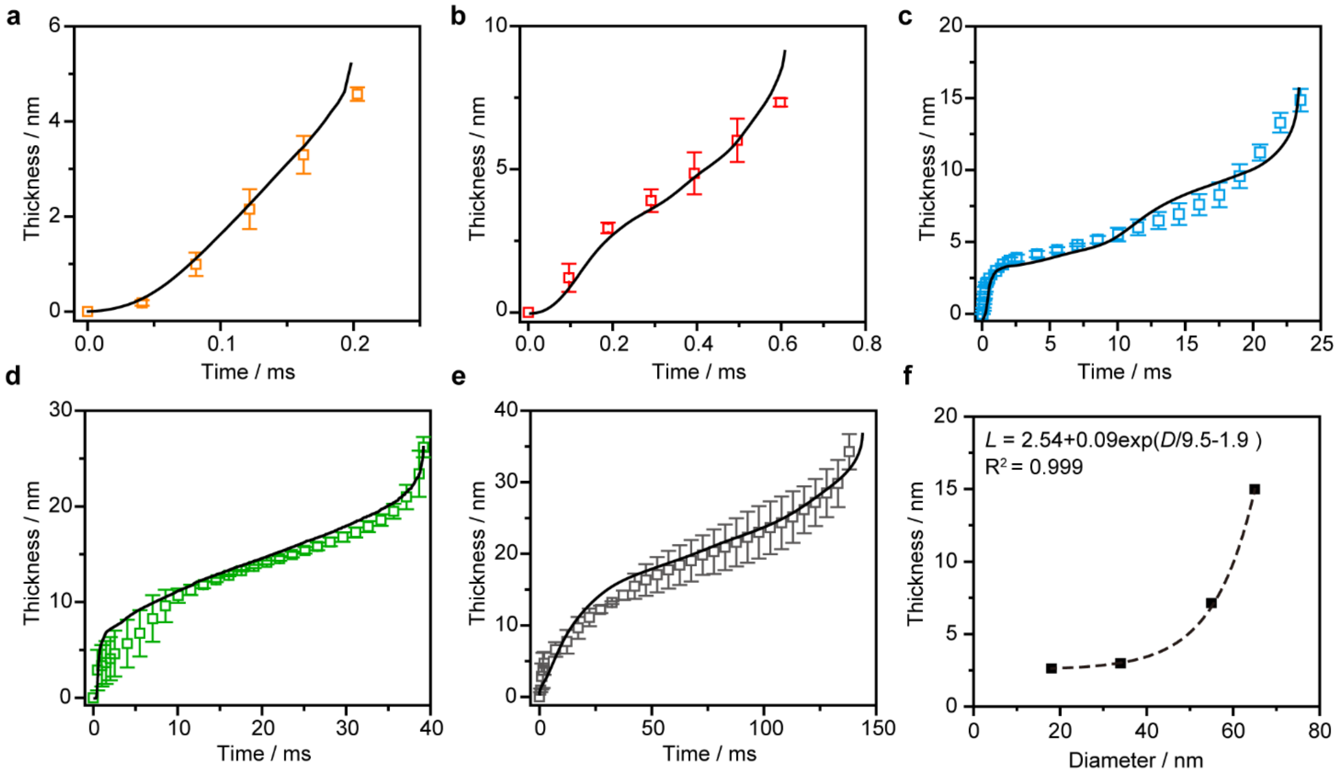

Supplementary Figure 14. Thickness of AgO film as a function of oxidation time. Plots of the experimental film thickness of oxidation time of AgNPs with different sizes (**a**) 10 nm, (**b**) 18 nm, (**c**) 34 nm, (**d**) 55 nm and (**e**) 65 nm vs the oxidation time. The black lines are the corresponding relations between the simulated film thickness and oxidation time. (**f**) Calibration curve of the critical AgO thickness vs the diameter of AgNPs. The black dotted line was fitted by equation (15), and the correlation coefficient is 0.999. Source data are provided as a Source Data file.

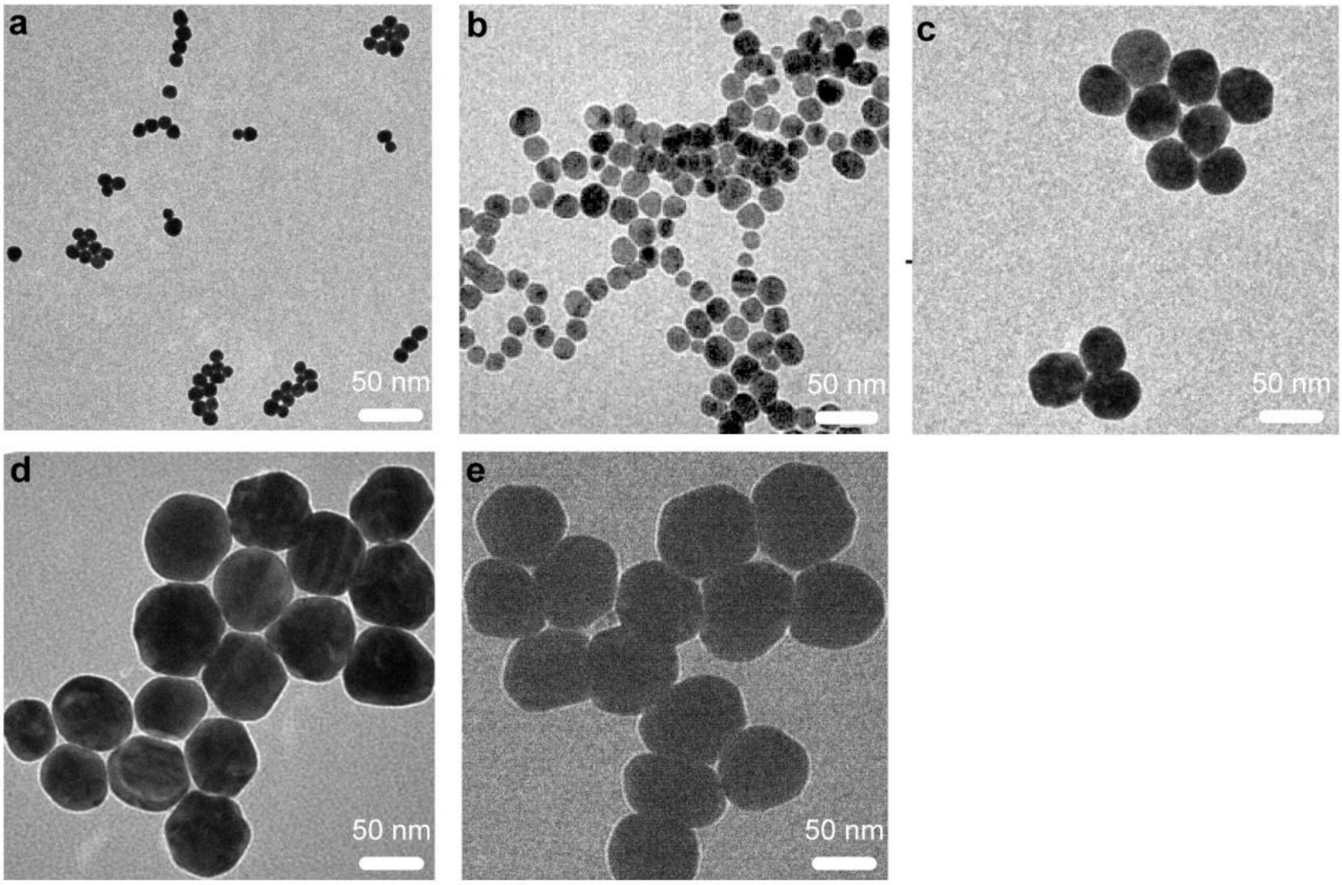

Supplementary Figure 15. TEM images of different sized AgNPs. (**a**) 10 nm, (**b**) 18 nm, (**c**) 34 nm, (**d**) 55 nm, and (**e**) 65 nm.

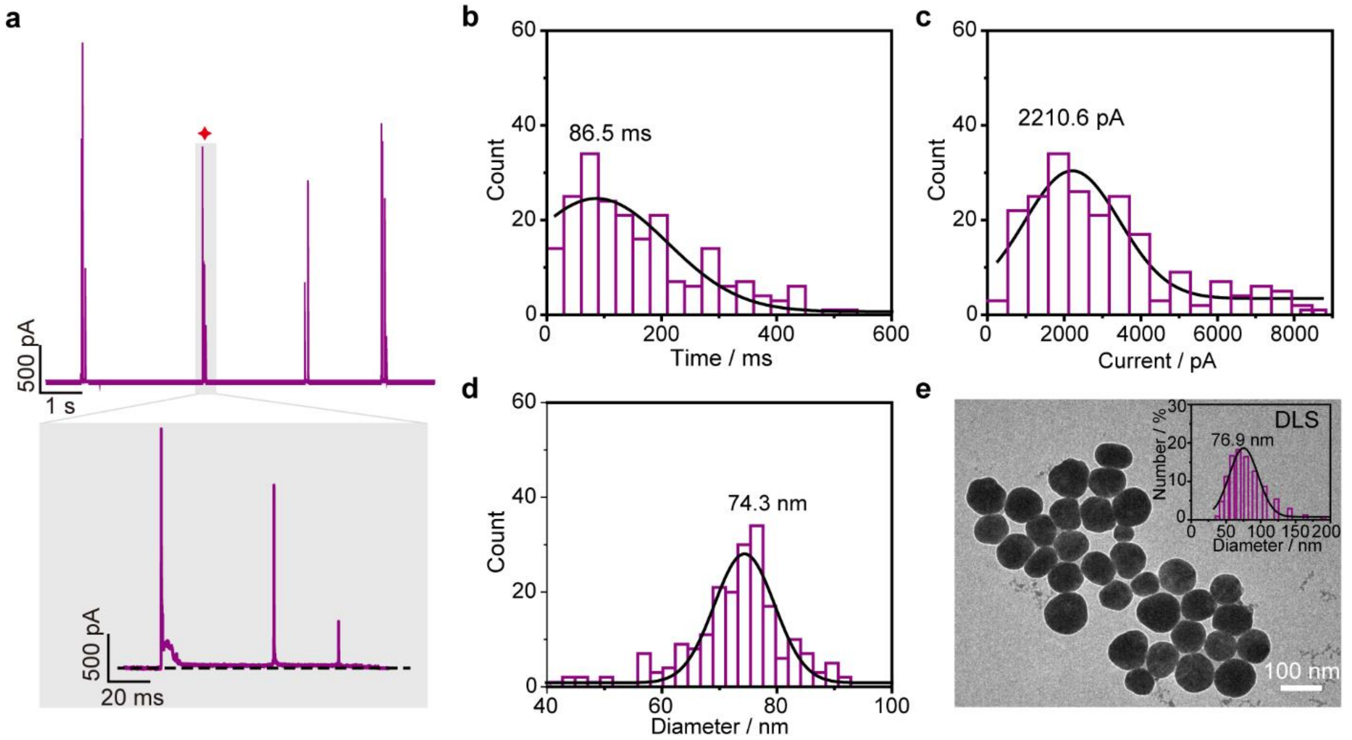

Supplementary Figure 16. Electrochemical oxidation of 75 nm AgNPs by surface-confined nano-collision strategy. (**a**) Chronoamperometric curves and representative current signals of individual AgNPs at +700 mV vs Ag/AgCl wire in alkaline solution at a Au UME (diameter 12.5 μm). Histograms of oxidation time (**b**), current (**c**) and diameter (**d)** of individual collision events, including Gaussian fits. (**e**) TEM image of AgNPs. Inset: DLS data of AgNPs. Source data are provided as a Source Data file.

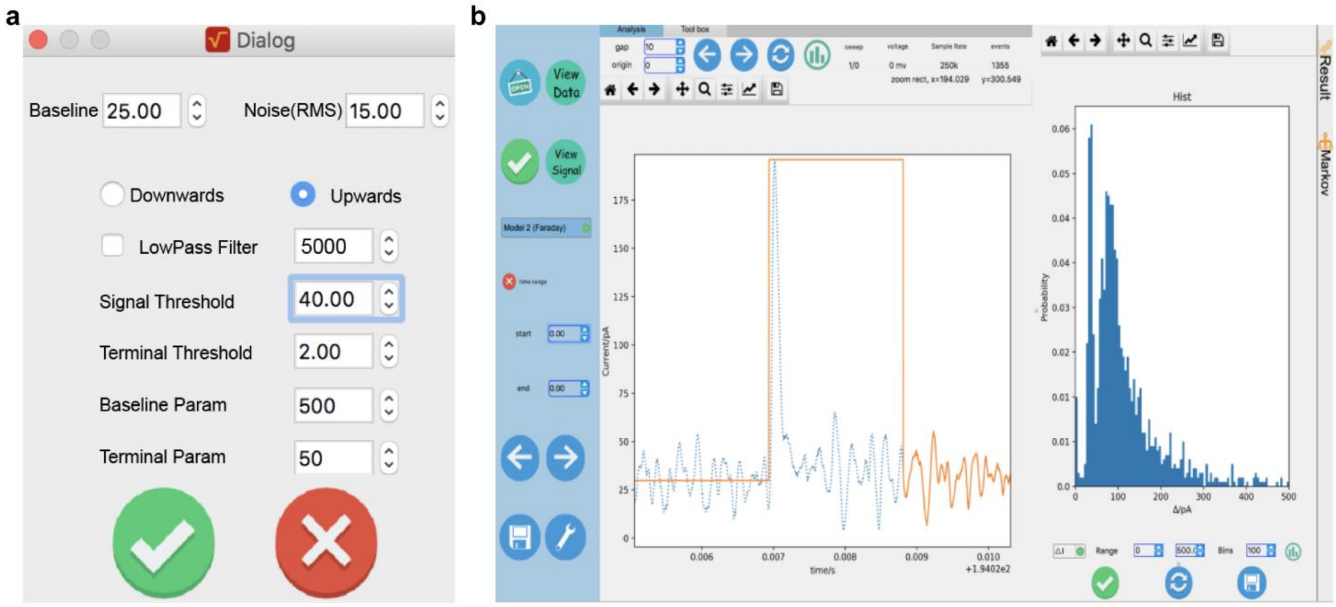

Supplementary Figure 17. Data analysis panel. (**a**) Operating interface of the self-designed PyNano software and the corresponding parameters. (**b**) Analysis of a typical signal. The signals selected by PyNano were automatically shown as the orange rectangular frame (right panel). The blue line represents the origin data and the orange horizontal line before the signal refers to the calculated baseline. Two vertical lines indicated the duration of the signal, while the region between the two horizontal lines represents the current amplitude of the signal. The distribution of current of collision events (right panel).

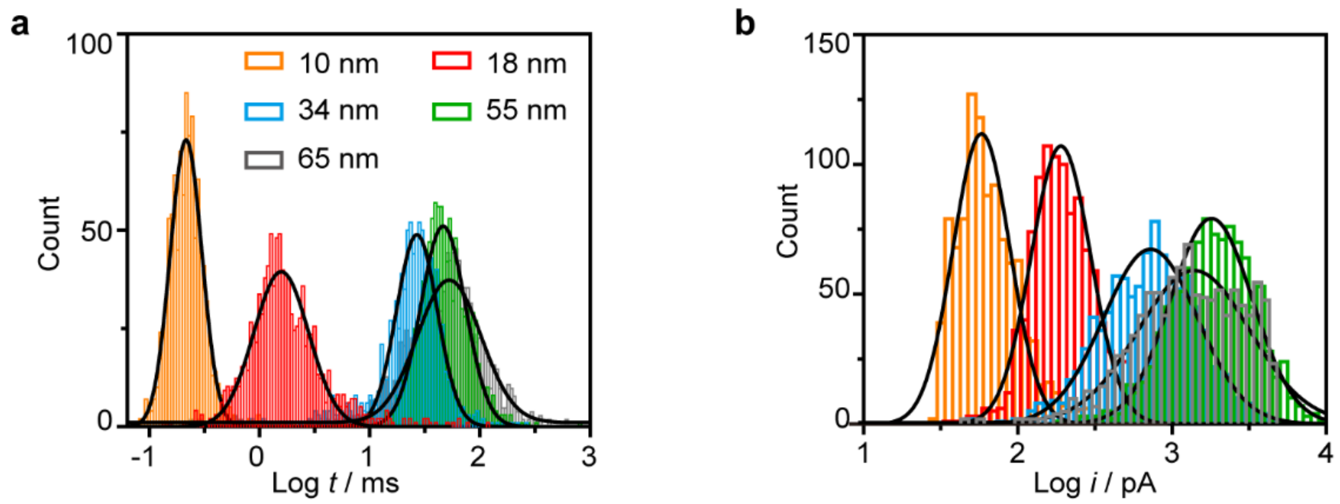

Supplementary Figure 18. Distribution of duration and current for individual collision events. Histograms of Log *t* (a) and Log *i* (b)for individual AgNPs at +600 mV vs Ag/AgCl wire in alkaline solution (pH = 11.4) at Au UME with Gaussian fits. Here, *t* and *i* represent duration time and the maximum current, respectively. The number of collisions in each histogram is at least 1,000. Source data are provided as a Source Data file.

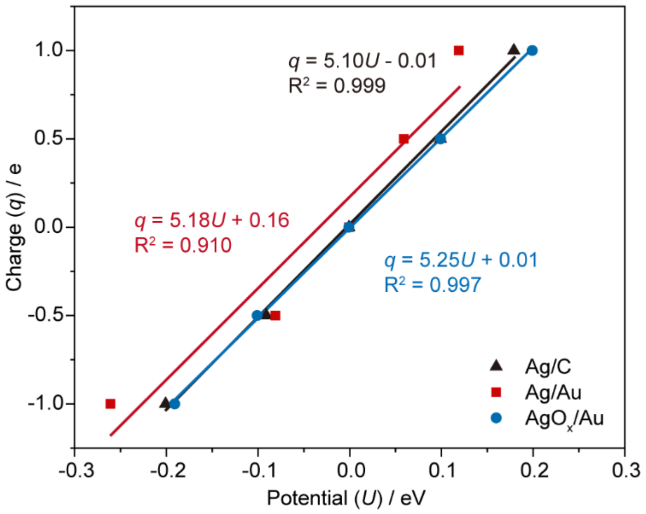

Supplementary Figure 19. Charge effect on DFT calculation. Calibration curves of the calculated electrode potential (U) with different charge values (q) for the Ag/C, Ag/Au and AgO_x/Au systems. The line has been fitted by regression analysis. Source data are provided as a Source Data file.

Supplementary Tables

Supplementary Table 1. Populations of coordination numbers for AgNP faces, edges, and vertexes in the icosahedral structure.

Type	Face	Edge	Vertex
Number of coordination numbers	9	8	6
Number of types	20	30	12

Supplementary Table 2. Calculated DFT adsorption energies of E_{ad}^{tot} and E_{ad} for the Ag/C, Ag/Au, and AgO_x/Au systems with different coordination numbers (n_c).

n_c	n_{ad}	E_{ad}^{tot} (eV)			E_{ad} (eV)		
		Ag/C	Ag/Au	AgO_x/Au	Ag/C	Ag/Au	AgO_x/Au
9	9	-0.91	-5.98	-9.75	-0.15	-0.66	-1.08
7	6	-1.08	-5.34	-7.01	-0.27	-0.89	-1.17
6	6	-1.12	-5.91	-7.04	-0.28	-0.98	-1.17
5	3		-3.31	-4.67		-1.10	-1.56
5	3	-1.06	-3.26	-4.74	-0.35	-1.09	-1.58
4	2	-1.04	-2.42	-3.80	-0.52	-1.21	-1.90
3	1	-0.83	-1.42	-2.47	-0.83	-1.42	-2.47

Supplementary Table 3. The diffusion-limited maximum current (i_{max}) of single AgNPs at different pH.

**pH**	**C_{OH-} (mol L^{-1})**	**i_{max} (pA)**
7.4	2.51×10^{-7}	0.05
8.6	3.98×10^{-6}	0.8
10.0	1.00×10^{-4}	19.5
11.4	2.51×10^{-3}	491.1
12.6	3.98×10^{-2}	7756.1

Supplementary Table 4. Calculated DFT adsorption energies of E_{ad} for the possible species in the solution on the NP surface and the electrode interface.

	E_{ad} (eV)					
	$H_2PO_4^-$	HPO_4^{2-}	Na^+	OH^-	Au	Ag
**Ag**	-0.02	0.51	-0.20	0.02	-5.98	/
**AgO_x**	2.14	4.37	-2.16	1.10	-9.75	/
**Au**	0.76	1.54	-0.81	0.86	/	-5.98
**C**	0.59	2.91	0.97	1.29	/	-0.91

Supplementary Table 5. Calculated charge values of q for the Ag/C, Ag/Au and AgO_x/Au systems with different electrode potential (U).

***U* (eV)**	***q* (e)**		
	**Ag/C**	**Ag/Au**	**AgO_x/Au**
-1.00	-0.20	-0.26	-0.19
-0.50	-0.09	-0.08	-0.10
0.00	0.00	0.00	0.00
0.50	0.10	0.06	0.10
1.00	0.18	0.12	0.20

Supplementary Table 6. Calculated DFT adsorption energies of E_{ad} for the citrate ion on the surface of AgNP and AgO NP.

E_{ad} (eV)	
citrate/Ag	citrate/AgO
-0.02	0.26

Supplementary Notes

Supplementary Note 1. Icosahedral structure of AgNPs.

To estimate the relation between the average coordination number (n_c) and particle radius (r), we approximated the AgNPs used in this work as 20 orderly arranged triangular-type (111) facets.[1] The icosahedral structure of AgNPs is shown in Supplementary Figure 1. The lowest-energy structures and the electronic properties of the icosahedrons were investigated based on a generalized gradient approximation. The populations of coordination numbers and atoms are given as functions of particle faces, edges and vertexs in icosahedral structure (Supplementary Table 1). The number of Ag layers (n) can be calculated by $n = r/d_{Ag}$, where d_{Ag} is the distance between two Ag atoms. Then, the relation between n_c and r can be achieved by the following expressions:

Total number of coordinations = $90(n-1)(n-2) + 240(n-1) + 72 = 90n^2 - 30n + 12$

Total number of atoms = $10(n-1)(n-2) + 30(n-1) + 12 = 10n^2 + 2$

Average number of coordinations (n_c) = $(90n^2 - 30n + 12)/(10n^2 + 2) = 9 - 3/n = 9 - 3d_{Ag}/r$

Supplementary Note 2. Interval time of two collision events at the C UME surface.

In single NP collision electrochemical measurements at a C UME, a spike with closely spaced clustering was clearly observed (Fig. 2e). As for the experimentally measured current trace, two situations may occur during the electrochemical process. First, the consecutive multistep collisions attributed to one AgNP electrooxidation event. Second, there is the simultaneous collisions of two or more particles with the electrode and being detected at the same time. To clearly understand the stochastic collision process of individual NPs, we employed Poisson distribution treatment to statistically study the datasets of collision signals. In this theoretical model, we defined an interval time window of two collision events (t) during which only one or zero single NP can collide at the C UME surface with 99 % confidence. That is, there is a 99 % collision probability of up to one AgNP that occur within the time interval. This indicates that the experimentally observed multi-spikes within this time interval widow are clustered as the multi-collisions of the same AgNP with the CUME. Under the assumption of independent collision events, the time interval window can be quantified by the probability theory of a Poisson distribution as follows by equation (1):[2]

$$P_n\,(t) = \frac{(\lambda \mathrm{t})^n}{n!} e^{-\lambda \mathrm{t}} \qquad (1)$$

where P is the probability and n is the number of collision events in a certain time interval t. λ is the average occurrence rate of such events, which can be calculated using a steady-state diffusion flux of the AgNPs to the C UME, J (s^{-1}), by equation (2):

$$J = 4D_{NP}C_{NP}r_{elec}N_A \qquad (2)$$

where C_{NP} is the concentration of AgNPs (37.8 pM), N_A is Avogadro's constant (6.02×10^{23} mol^{-1}), and r_{elec} is the radius of the C UME (3.5 μm). The diffusion coefficient of an AgNP (D_{NP}) can be determined from the Stokes-Einstein equation, equation (3):

$$D_{NP} = \frac{k_\mathrm{B}\mathrm{T}}{6\pi\eta \mathrm{r_{NP}}} \qquad (3)$$

where k_B is the Boltzmann constant (1.38×10^{-23} J K^{-1}), T is the absolute temperature (298 K), η is the

solution viscosity (8.94×10^{-4} P s) and r_{NP} is the radius of AgNP (17 nm). The diffusion coefficient of the AgNPs according to equation (3) is 1.44×10^{-11} m^2 s^{-1}. Herein, the calculated flux J is the average occurrence rate λ in the Poisson distribution equation. As a result, the theoretical interval time of two collision events of individual AgNPs is 32 ms at a 7.0 μm diameter collecting electrode. Therefore, the theoretical results suggested that it was unlikely that multiple collisions of different particles occurred on a mili-second timescale, the experimental observation of clusters of close, consecutive current spikes can rather be attributed to the oxidation of a single AgNP *via* a series of stages, during each of which the NP is partially oxidized.

Supplementary Note 3. DPV for electrochemical oxidation of AgNPs in alkaline media.

DPV was subsequently used for electrochemical measurements with AgNPs modified Au electrode in alkaline media (15 mM PB and 10 mM NaOH). These voltammetry scans started from -200 mV to +800 mV *vs* Ag/AgCl wire reference electrode and platinum counter electrode set a scan rate of 20 mV s^{-1}. As shown in Supplementary Figure 8, four oxidative peaks (A1-A4) are apparent at about -0.01, 0.09, 0.25, and 0.53 V *vs* Ag/AgCl wire reference. The anodic peak referred to A1–A3 and A4 in the DPVs coincide with those reported in previous reports and associated with the formation of Ag_2O and AgO).[3] According to a literature review,[4-6] via the adsorption of OH^- and desorption and diffusion of soluble $[Ag(OH)_2]^-$, A1 the first weak anodic peak might be assigned to the electro-dissolution of Ag to $[Ag(OH)_2]$-:

$$\mathrm{Ag} + 2\mathrm{OH}^- \longrightarrow [\mathrm{Ag(OH)_2}]^-_{ads} + \mathrm{e}^- \qquad (4)$$

$$[\mathrm{Ag(OH)_2}]^-_{ads} \longrightarrow [\mathrm{Ag(OH)_2}]^-_{aq} \qquad (5)$$

The second anodic current peak (A2) could be attributed to the formation of a monolayer of Ag_2O at the electrode surface on account of the precipitation of $[Ag(OH)_2]^-$ from its supersaturated solution as confirmed by optical studies.[4,7] The rate of anodic dissolution and the formation of $[Ag(OH)_2]^-$ were reduced by the formation of a monolayer of Ag_2O[6], which well correspond to the experimental results. The third peak A3 could be attributed to forming a multilayer of Ag_2O by thickening of the basal monolayer[4,5] through the following reaction:

$$2\mathrm{Ag} + 2\mathrm{OH}^- \longrightarrow \mathrm{Ag_2O} + \mathrm{H_2O} + 2\mathrm{e}^- \qquad (6)$$

The anodic peak A4 is ascribed to the electrooxidation of Ag_2O and the formation of AgO through the following reaction:[3]

$$\mathrm{Ag_2O} + 2\mathrm{OH}^- \longrightarrow 2\mathrm{AgO} + \mathrm{H_2O} + 2\mathrm{e}^- \qquad (7)$$

Furthermore, the formation of Ag to AgO directly occurs within the potential range of peak A4:[8-11]

$$\mathrm{Ag} + 2\mathrm{OH}^- \longrightarrow \mathrm{AgO} + \mathrm{H_2O} + 2\mathrm{e}^- \qquad (8)$$

These results revealed one-electron oxidation and two-electron oxidation that occurred in the process of oxidation of silver at the different applied potential.

Supplementary Note 4. Characterization of AgO formation.

As shown in Supplementary Figure 11, the binding energies of $Ag3d_{5/2}$ of AgO, Ag_2O and Ag^0 are 367.4, 367.8 and 368.2 eV, respectively, which are consistent with the previously reported values.[12-14] Moreover, the oxidation products mainly contain the Ag_2O and a small amount Ag^0 with a bias potential of +0.4 V vs Ag/AgCl, while major constituents of the sample at the potential of +0.6 V vs Ag/AgCl are the AgO. Additionally, we also used X-ray Diffraction (XRD) to characterize the 34 nm AgNPs dropped indium-tin oxide (ITO) electrode after anodic polarization for 1 h at +600 mV vs Ag/AgCl in alkaline media (15 mM PB and 10 mM NaOH). The diffraction peaks observed around 2θ = 30-60° can be indexed to AgO with JCPDS No. 74-1743 (Supplementary Figure 11c), further demonstrating the formation of silver oxide in our system.[15,16] These results confirmed the two-electron oxidation of AgNPs in alkaline media at +0.6 V vs Ag/AgCl wire.

Supplementary Note 5. pH effect on electrochemical oxidation of individual 34 nm AgNPs.

To understand the underlying mechanism of the pH effect to the AgNPs oxidation in alkaline solution, we further investigated the electrochemical behaviors of 34 nm AgNPs in diameter at different pH as a model (Supplementary Figure 12). The current signals with short oxidation time and high maximum current were observed in neutral solution (pH = 7.4), which was attributed to the fast oxidation kinetics for AgNPs dissolving to Ag^+. The integrated charge was consistent with the completely oxidative charge of single 34 nm AgNPs involving 1e^- transfer process. When pH of the electrolyte varying from 7.4 to 10.0, the current amplitude significantly decreased while the oxidation time slightly elongated. Accordingly, the integrated charge decreased slowly. We proposed that the slow rates of AgNPs oxidation were attributed to the limited amount of OH^- and the formation of insoluble silver oxide on the particle surface, which partly passivated the oxidation process.[17] In the case of the diffusion-limited oxidation, the flux of hydroxyl ions to the surface of AgNPs played a dominant role, which substantially influenced the oxidation current trace of individual NPs. The current transients ($i(t)$) can be described as the diffusion-limited flux to a sphere with a shrinking radius due to the continuous consumption of AgNP by the electro-oxidation reaction,[17,18] which is given by:

$$i(t) = 4\pi nF\, ln\, 2\, D_0 C_0 \sqrt{r_0^2 - \frac{2ln2N_A D_{\mathrm{OH^-}} C_0 m_{atom}}{\rho} t} \qquad (9)$$

where D_0 is the diffusion coefficient of hydroxyl ions (6.8×10^{-9} m^2·s^{-1}), C_0 is the bulk hydroxyl ion concentration, and r_0 is the radius of AgNP (17 nm). m_{atom} and ρ are the atomic mass and the density of silver, respectively. It can be expected from equation (9) that the maximum current (i_{max}) of single AgNP linearly with the concentration of hydroxyl ion, when t = 0. As shown in Supplementary Table 3, the diffusion-limited maximum current of single AgNPs was obviously beneath the experimentally measured results, when pH≤ 10, demonstrating the decreased current because of the deficiency of hydroxyl ions.

When the pH value further increased, the maximum current of AgNPs with the same size basically unchanged in the sufficient concentration of OH^-, evidencing the electrochemical reaction rate-limited processes of AgNPs. While the oxidation time and integrated charge dramatically increased due to the enhanced attractive interaction between the silver oxide and the Au UME. Notably, the integrated charge of individual AgNPs at pH = 11.4 and 12.6 was almost twice that of neutral solution (pH = 7.4), resulting from the $2e^-$ complete oxidation of Ag to form AgO.[19]

Supplementary Note 6. Thickness of AgO film.

By analyzing the integrate charge (q) of the initial spike with different particle sizes (diameter: 10 nm, 18 nm, 34 nm, 55 nm and 65 nm), we obtained the ratio of the average faradaic charge from the initial spike to the total charge (Q) (Supplementary Figure 13). The results illustrate that the correlation between the ratio and the size is approximately exponential relationship and could be well fitted by equation (10):

$$\frac{q}{Q} = 2.1 + e^{-\frac{d}{17.4}} \qquad (10)$$

where d is the diameter of AgNPs. In this work, we assume that all NPs are spherical, and the silver oxide layers form from the outside surface to the inside of AgNPs. Therefore, the AgO shell thickness (L) can be calculated through equation (11):

$$L = r - \sqrt[3]{\frac{3(Q - q(t))\mathrm{Ar}}{4\pi n \mathrm{F} \rho}} \qquad (11)$$

where r is the radius of the AgNPs, $q(t)$ is the integrate charge at any time, n is the number of electrons transferred per Ag atom, Ar is the atomic molecular mass of Ag (107.9 g mol^{-1}), F is Faraday's constant, and ρ is the density of Ag (10.5×10^6 g m^{-3}). According to equation (11), the AgO shell thickness of AgNPs with the diameters of 10, 18, 34, 55, and 65 nm corresponding to the integrated charge of the initial spike are 1.78, 1.51, 1.11, 0.73 and 0.58 nm, respectively. Therefore, the average numbers of oxidized Ag atomic layers during the first subpeak of individual collision are 5.4, 4.6, 3.4, 2.2 and 1.8 for five sized AgNPs.

To better understand this interesting collision/oxidation behavior in greater detail, we further investigated the AgO film growth kinetics on the surface of different sized AgNPs with oxidation time using a dynamic Monte Carlo simulation (Supplementary Figure 14). Considering $2e^-$ oxidation in alkaline media (pH = 11.4) at the potential of +600 mV vs Ag/AgCl wire, the Faradic current contributing to particle oxidation at any time $i(t)$ during the collision event of an AgNP is given by:

$$i(t) = \frac{\partial Q(t)}{\partial t} = -\frac{8\pi r_i^2 F \rho \partial r_i}{\mathrm{Ar}} \frac{\partial r_i}{\partial t} \qquad (12)$$

where r_i is the radius of an AgNP at any time. By integrating the Faraday current, we can obtain the total charge associated with AgNP oxidation:

$$Q = \int_0^{t_f} i(t)dt = \int_0^{t_f} -\frac{2F\rho}{\mathrm{Ar}} dV(t) = \frac{8\pi r_0^3 F\rho}{3\mathrm{Ar}} \quad (13)$$

where t_f is the entire oxidation time of an AgNP, r_0 is the initial size of AgNPs. According to equation (13), we can calculate r_0 and extract the radius of an AgNP at any time:

$$r_i = \sqrt[3]{\frac{3\mathrm{Ar}\int_{t_i}^{t_f} i(t)dt}{8\pi F\rho}} \quad (14)$$

The thickness of AgO can be calculated by $L = r_0 - r_i$. As shown in Supplementary Figure 13a-e, the black lines are the relations between the simulated AgO film thickness on the surface of AgNP and oxidation time, which agree well with the experimental results (plots in Supplementary Figure 14a-e). The AgO thickness of various sized AgNPs increased quickly at first and then thicken slowly with a substantial inflection point, corresponding to the critical AgO thickness for the transition from fast to slow oxidation. Except for 10 nm diameter AgNPs, the AgO film thicken continuously at a steady rate without inflection point (Supplementary Figure 14a). Differences in the AgO formation process could be interpreted from the level of mass transfer of Ag^+. Since the slow release of Ag^+ in the presence of the low solubility of silver oxide, the smaller particles with the larger surface area to mass ratio, resulting in the fast mass transfer. Based on the analysis of AgO thickness formation curves, the relation between the critical AgO thickness and diameter of AgNPs can be described by equation (15):

$$L_c = 2.54 + 0.09e^{(\frac{d}{9.5}-1.9)} \quad (15)$$

where L_c is the critical AgO thickness on the surface of AgNPs for the transition from fast to slow oxidation. Supplementary Figure 14f exhibited a strong size-dependent critical AgO thickness, which grows exponentially with the increasing diameter of AgNPs. Notably, the critical AgO thicknesses of various sized NPs are inconsistent with the first subpeak of an individual collision, which could be attributed to a

combination effect of size shrinking and solubility-limited kinetic. In the initial oxidation process, the size shrinking dominated the oxidation current, generating amount of Ag^{+}. Subsequently, Ag^{+} reacted with OH^{-} and then the solubility-limited kinetic took charge.

Supplementary Note 7. Data acquisition and analysis.

Data were acquired at a sampling rate of 100 kHz by using an A/D converter and home-designed PC running software. The data analysis was performed. The data in nano-collision experiments were analyzed using python based self-designed software named PyNano (https://github.com/decacent/PyNano). Each file of data in the "abf" format could be imported into PyNano directly by pushing the button of "OPEN", and we could further set parameters such as base current and minimum current amplitudes (Supplementary Figure 17a). In the analysis of collision events of the AgNPs mixtures with the diameter of 10 nm and 18 nm in alkaline media, the value of baseline was chosen to be 25 pA and signals with current amplitudes of less than the threshold value of 40 pA were ignored, as they were evaluated considering the signal noise. Moreover, we used 500 points to judge the baseline before the beginning of a signal and 50 points to estimate the end of the signal (the deviation between these points and baseline less than 2 pA). Through PyNano, signals could be classified based on their shapes, current amplitudes, and duration time consecutively (Supplementary Figure 17b, left panel). In addition, all parameters could be optimized to obtain more accurate signals. PyNano could rapidly select signals automatically one by one, and the distribution of duration time, current and integrated charge can directly be plotted in software systems (Supplementary Figure 16b, right panel) within a few minutes. Finally, all extracted parameters could be output into a Microsoft Excel file for further analysis.

Supplementary Note 8. Histograms of Log *t* and Log *i* of individual collision events.

As shown in Supplementary Figure 18, these excellent-resolved current traces yielded clearly distinguishable histograms of Log *t* and Log *i* using a python based self-designed software within several minutes. Gaussian peak values were assigned to the AgNPs of various sizes, with highly sensitive to realize sizes discrimination of NPs from 10 to 65 nm in diameter. The statistical results of Log *t* from the analysis of over 1000 collisions of each sample presented in Supplementary Figure 18a show five sharp distributions which were corresponding to 0.2 ± 0.1 ms, 1.6 ± 0.5 ms, 26.3 ± 2.2 ms, 46.5 ± 3.0 ms, and 52.5 ± 3.5 ms for 10 nm, 18 nm, 34 nm, 55 nm and 65 nm AgNPs in diameter, respectively. Accordingly, Log *i* of the current signals also exhibit fiver distributions with partially overlap at 1.7 ± 0.2, 2.3 ± 0.2, 2.8 ± 0.3, 3.3 ± 0.2, and 3.2 ± 0.3, respectively (Supplementary Figure 18b). These results show that the chemical confined nano-collision strategy in alkaline could clearly reveal the size-dependent electrochemical oxidation behaviors during the dynamic collision process, providing multi-parameter accurate analysis of NPs.

Supplementary Note 9. Effects of electrolyte, charge and capping agent on DFT calculation.

1) Electrolyte effect

In this study, we investigated the electrochemical responses of individual AgNPs collisions at +600 mV vs Ag/AgCl (i) in neutral solution (25 mM PB, pH = 7.4) at a C UME; (ii) in neutral solution (25 mM PB, pH = 7.4) at a Au UME; and (iii) in alkaline media (15 mM PB and 10 mM NaOH, pH = 11.4) at a Au UME. Considering the presence of species in solution, we calculated their adsorption energies of the possible species on the NPs surface and electrode interface by the first principle DFT calculations. As shown in Supplementary Table 4, the other species except for Na^+ were found to have the weak affinity at the NP surface and electrode interface. For Na^+ ion, we can see that the competitive adsorption energies of the NPs on the electrode interface are much more exothermic than that of Na^+ on Ag, AgOx and Au (*Na/Ag* vs *Ag/C*, Na/AgO_x vs AgO_x/Au and *Na/Au* and *Ag/Au* are -0.20 vs -0.91 eV, -2.16 vs -9.75 eV and -0.81 vs -5.98 eV, respectively). Moreover, together with the low concentration of the species (<<1 mol L^{-1}) in the solution, the coverage or probability of these species on the surface would expect to be very low. Therefore, the adsorption of the possible species in the solution on the NP surface and the electrode interface could be ignored in our DFT calculation.

2) Charge effect

Considering the inclusion of electrons on the electrode during the electrochemical processes, we introduced two kind of methods for correcting the charge effects in this DFT calculations.

Firstly, we employed a simple electrode potential-corrected energy method, which is widely used in the electrochemical reaction proposed by Nørskov,[20] to investigate the charge influence to adsorption energy. In addition to the normal adsorption energy obtained by standard DFT calculations, the adsorption energy change introduced by the change in the electrode potential can be realized through shifting the energy level by $-neU$, where n is the electron transfer number for a given reaction. Considering the same applied potential

at +600 mV vs Ag/AgCl used in our electrochemical measurement, the electrode potential effect was normally ignored due to the same correction value.

On this basis, we further estimated the electrode potential dependent effect to the adsorption energy using another charge correction method.[21] In this correction, the electrode surface are assumed to be a plane-parallel capacitor, and then corrected the value by the energy of $1/2CU^2$. By adding the different charges on the electrode surface, we calculated the potential changes of the systems by the DFT calculations (Supplementary Table 5). By the slop of q-U relations, we estimated the capacitances of the electrode surfaces ($C = \Delta q/\Delta U$, Supplementary Figure 19). Clearly, we can see that the capacitances (C) are very similar. To make an equivalent comparison, the electrical potential at the same distance of NP on the electrode surface was used to describe the correction value. Although the electric potential U drops sharply with the increasing distance X according to Poisson Boltzmann equation, the electric potential U is still identical at a certain distance X on the electrode|solution interface.[22] As a result, the correction values of the electric potential ($1/2CU^2$) were usually approached to be similar for the Ag/C, Ag/Au and AgO_x/Au systems. For example, at the electric potential of +600 mV vs Ag/AgCl (corresponding to the state that the NP adsorbed on the electrode surface at the distance $X = 0$), the correction values $1/2CU^2$ are 0.918, 0.932 and 0.945 eV for the Ag/C, Ag/Au and AgO_x/Au systems, respectively. Therefore, the potential dependent effect was negligible in our simulations due to the similar correction value. Two kinds of methods have both demonstrated that the correction values of the applied potential were usually approached to be similar, and thus charge effects were also ignored in our simulations.

3) Capping effect

In this work, citrate-capped AgNPs were used for the entire electrochemical experiment. We further investigated the capping agent effect of citrate ion to DFT results. According to the obtained results by the first principle DFT calculations, the adsorption energies of the citrate ion on the AgNP and AgO NP surface

are -0.02 eV and 0.26 eV, respectively (Supplementary Table 6). Compared with the adsorption energies of NP on the electrode surface (Supplementary Table 2), the interactions between citrate ion and NP were significantly weak.

Moreover, due to the continuously refreshed particle surface during the electrochemical oxidation of AgNPs and the trace citrate ion in the solution, the probability of citrate ion back to NPs surface would expect to be very low. Taken together, we did not take the effect of the surface capping agent (citrate) into consideration for adsorption energy in this DFT calculation.

Supplementary Reference

1. Zhang, Q., Xie, J., Yang, J. & Lee, J. Y. Monodisperse icosahedral Ag, Au, and Pd nanoparticles: Size control strategy and superlattice formation. *ACS Nano* **3**, 139–148 (2009).

2. Xie, R., Batchelor-McAuley, C., Young, N. P. & Compton, R. G. Electrochemical impacts complement light scattering techniques for in situ nanoparticle sizing. *Nanoscale* **11**, 1720–1727 (2019).

3. Abd El Rehim, S. S., Hassan, H. H., Ibrahim, M. A. M. & Amin, M. A. Electrochemical behaviour of a silver electrode in NaOH solutions. *Monatshefte für Chemie / Chem. Mon.* **129**, 1103–1117 (1998).

4. Tilak, B., Perkins, R., Kozlowska, H. & Conway, B. Impedance and formation characteristics of electrolytically generated silver oxides-I formation and reduction of surface oxides and the role of dissolution processes. *Electrochim. Acta* **17**, 1447–1469 (1972).

5. Ambrose, J. & Barradas, R. G. The electrochemical formation of Ag_2O in KOH electrolyte. *Electrochim. Acta* **19**, 781–786 (1974).

6. Tilak, B. V., Perkins, R. S., Kozlowska, H. A. & Conway, B. E. Impedance and formation characteristics of electrolytically generated silver oxides—I formation and reduction of surface oxides and the role of dissolution processes. *Electrochim. Acta* **17**, 1447–1469 (1972).

7. Amlie, R. F., Honer, H. N. & Ruetschi, P. The Voltage increase of the cuprous chloride electrode by the addition of sulfur. *J. Electrochem. Soc.* **112**, 1073 (1965).

8. Giles, R. D. & Harrison, J. A. Potentiodynamic sweep measurements of the anodic oxidation of silver in alkaline solutions. *J. Electroanal. Chem. Interfacial Electrochem.* **27**, 161–163 (1970).

9. Burstein, G. T. & Newman, R. C. Anodic behaviour of scratched silver electrodes in alkaline solution. *Electrochim. Acta* **25**, 1009–1013 (1980).

10. Hoar, T. P. & Dyer, C. K. The silver/silver-oxide electrode—I. Development of electrode by slow ac cycling. *Electrochim. Acta* **17**, 1563–1584 (1972).

11. Salvarezza, R. C., Gómez Becerra, J. & Arvia, A. J. Kinetics and mechanism of the silver (I) oxide

to silver (II) oxide layer electrooxidation reaction. *Electrochim. Acta* **33**, 1753–1759 (1988).

12. Gao, X.-Y. *et al.* Study of structure and optical properties of silver oxide films by ellipsometry, XRD and XPS methods. *Thin Solid Films* **455**, 438–442 (2004).

13. Weaver, J. F. & Hoflund, G. B. Surface Characterization Study of the Thermal Decomposition of AgO. *J. Phys. Chem.* **98**, 8519–8524 (1994).

14. Waterhouse, G. I. N., Bowmaker, G. A. & Metson, J. B. Oxidation of a polycrystalline silver foil by reaction with ozone. *Appl. Surf. Sci.* **183**, 191–204 (2001).

15. Waterhouse, G. I. N., Bowmaker, G. A. & Metson, J. B. The thermal decomposition of silver (I, III) oxide: A combined XRD , FT-IR and Raman spectroscopic study. *Mater. Lett.* **47**, 319–323 (2001).

16. Behpour, A. S. M. Synthesis and characterization of AgO nanostructures by precipitation method and its photocatalyst application. *J. Mater. Sci. Mater. Electron.* **27**, 1191–1196 (2016).

17. Kätelhön, E., Tanner, E. E. L., Batchelor-McAuley, C. & Compton, R. G. Destructive nano-impacts: What information can be extracted from spike shapes? *Electrochim. Acta* **199**, 297–304 (2016).

18. Krause, K. J. *et al.* The influence of supporting Ions on the electrochemical detection of individual silver nanoparticles: Understanding the shape and frequency of current transients in nano-impacts. *Chem. - A Eur. J.* **23**, 4638–4643 (2017).

19. Amlie, R. F. & Rüetschi, P. Solubility and stability of silver oxides in alkaline electrolytes. *J. Electrochem. Soc.* **108**, 813–819 (1961).

20. Nørskov, J. K. *et al*. Origin of the overpotential for oxygen reduction at a fuel-cell cathode. *J. Phys. Chem. B* **108**, 17886-17892 (2004).

21. Skúlason, E. *et al*. Density functional theory calculations for the hydrogen evolution reaction in an electrochemical double layer on the Pt (111) electrode. *Phys. Chem. Chem. Phys.* **9**, 3241-3250 (2007).

22. Tschulik, K., Cheng, W., Batchelor-McAuley, C., Murphy, S., Omanović, D. & Compton, R. G. Non-invasive probing of nanoparticle electrostatics. *ChemElectroChem* **2**, 112-118 (2015).
